# Feasibility of uniportal thoracoscopic sublobar resection without chest tube drainage: a retrospective cohort study

**DOI:** 10.3389/fonc.2026.1773894

**Published:** 2026-06-18

**Authors:** Xiaozun Yang, Bin Hu

**Affiliations:** Department of Thoracic Surgery, Sichuan Clinical Research Center for Cancer, Sichuan Cancer Hospital & Institute, Sichuan Cancer Center, University of Electronic Science and Technology of China, Chengdu, Sichuan, China

**Keywords:** air leakage test, feasibility, safety, thoracoscopic sublobar resection, tubeless strategy

## Abstract

**Background:**

Tubeless strategy of thoracoscopic surgery has become a new topic in recent years. This study aimed to investigate the feasibility of no chest tube drainage compared with chest tube drainage in patients who underwent uniportal thoracoscopic sublobar resection.

**Methods:**

Patients who underwent uniportal thoracoscopic sublobar resection with or without chest tube drainage were included in this retrospective cohort study. They were required to have a negative intraoperative air leakage test. Data regarding perioperative outcomes, postoperative complications, postoperative pain visual analogue scale (VAS) score at rest, and patients’ satisfaction were collected.

**Results:**

A total of 60 patients without chest tube drainage served as the tubeless group, while another 60 age/sex-matched patients with chest tube drainage served as the chest tube group. Pneumothorax occurred in only one (1.7%) patient in the tubeless group, which was not significantly different from 0 (0.0%) patient in the chest tube group (*P =* 0.500). This patient was further readmitted and underwent conversion to chest tube drainage. No patient experienced other postoperative complications such as pleural effusion and pulmonary infection in either group. The operational time (*P* < 0.001), perioperative blood loss (*P =* 0.007), total hospital stay (*P =* 0.001), and postoperative hospital stay (*P* < 0.001) were lower in the tubeless group than in the chest tube group. The pain VAS score was decreased in the tubeless group compared with the chest tube group on postoperative day (POD) 1 (*P =* 0.003) and 2 (*P =* 0.020), but not changed on POD 3 (*P =* 0.200). Moreover, patients’ satisfaction scale (*P =* 0.410) and overall satisfaction rate (*P =* 0.375) were not different between the two groups. After adjustment using multivariable regressions, tubeless versus chest tube was independently correlated with a lower operational time (*P* < 0.001), total hospital stay (*P =* 0.003), postoperative hospital stay (*P* < 0.001), and pain VAS score on POD1 (*P =* 0.009) and POD2 (*P =* 0.042) but was not independently associated with perioperative blood loss (*P =* 0.110), pain VAS score on POD3 (*P =* 0.348), pneumothorax presence (*P =* 0.997), or overall satisfaction (*P =* 0.553).

**Conclusion:**

Uniportal thoracoscopic sublobar resection without chest tube drainage may be feasible in the selected, low-risk patients without intraoperative air leakage. However, further prospective studies with standardized outcome capture are still needed for validations.

## 1 Introduction

Video-assisted thoracoscopic surgery (VATS) is a common technique widely applied in lung surgeries and is associated with low invasiveness and few complications (1–4). Recent improvements have been made in VATS technology, such as the transformation from multiportal to uniportal approaches, evolution to robot-assisted surgery, tubeless VATS, and image-guided techniques (5–8). After thoracoscopic lung surgeries, chest tube drainage is a common method that involves the drainage of air and blood from the cavity (9–11).

Along with the progress of VATS technology and instruments, minimized incisions and restricted resection have been introduced, which largely reduce postoperative bleeding and air leakage in some types of lung surgeries, such as thoracoscopic wedge resection; in these cases, a tubeless strategy in which chest tube drainage is omitted may be available, which reduces postoperative pain and infection, improves wound recovery and shortens the hospital stay (12–15).

Earlier studies reported an awareness of the potential risk of postoperative pneumothorax and/or pleural effusion when a tubeless strategy of VATS was used (16, 17). Encouragingly, recent studies have reported good tolerance with comparable pneumothorax or pleural effusion incidence between tubeless strategy and chest tube drainage in selective-VATS-treated patients screened by preoperative condition, surgery type, lesion size, operation time, and especially the air leak test (12, 14, 15). Moreover, a recent Chinese regional expert consensus proposed the consideration of thoracoscopic sublobar resection without chest tube drainage in selected patients who had specific primary surgeries, operational time ideally within 2 h, and normal cardiopulmonary and renal functions (7). Clinically, thoracoscopic sublobar resection, including segmentectomy and wedge resection, allows the preservation of more lung function, less invasiveness, and fewer complications, increasing the likelihood of a tubeless strategy for VATS (18). To date, only a few studies have reported the application of the tubeless strategy of VATS in Chinese patients, and these studies have focused mainly on wedge resection. With respect to the tubeless strategy of VATS in Chinese patients, which focuses on thoracoscopic sublobar resection including segmentectomy and wedge resection, relevant evidence is limited.

Therefore, this study aimed to investigate the feasibility of no chest tube drainage compared with chest tube drainage in patients who underwent uniportal thoracoscopic sublobar resection.

## 2 Materials and methods

### 2.1 Subjects and groups

Patients who underwent uniportal thoracoscopic sublobar resection with or without chest tube drainage between January 2023 and December 2024 were included in this retrospective cohort study. The main inclusion criteria were as follows: (1) patients who underwent uniportal thoracoscopic sublobar resection, (2) patients aged ≥18 years, (3) patients with or without chest tube drainage after surgery, and (4) patients with accessible data that could be retrieved for the study use. The main exclusion criteria were as follows: (1) patients underwent major lung surgery such as thoracotomy and lobectomy, (2) patients underwent bilateral lung surgery, and (3) patients had a history of cardiothoracic surgery before the uniportal thoracoscopic sublobar resection. This study was approved by the Ethics Committee of our institution, and the patients or their families provided informed consent. The patients who omitted chest tube drainage after surgery were regarded as the tubeless group in this study, while the patients who received chest tube drainage after surgery were regarded as the chest tube group. Patients in the chest tube group were 1:1 age- and sex-matched to those in the tubeless group, with age matching based on the maximum allowable difference method and sex matching based on the frequency matching method.

### 2.2 Brief procedures

After general anesthesia was induced, single-lung ventilation and sublobar resection were performed cautiously according to routine standards. Sublobar resection includes two types of surgeries: segmentectomy and wedge resection.

**Figure 1:**
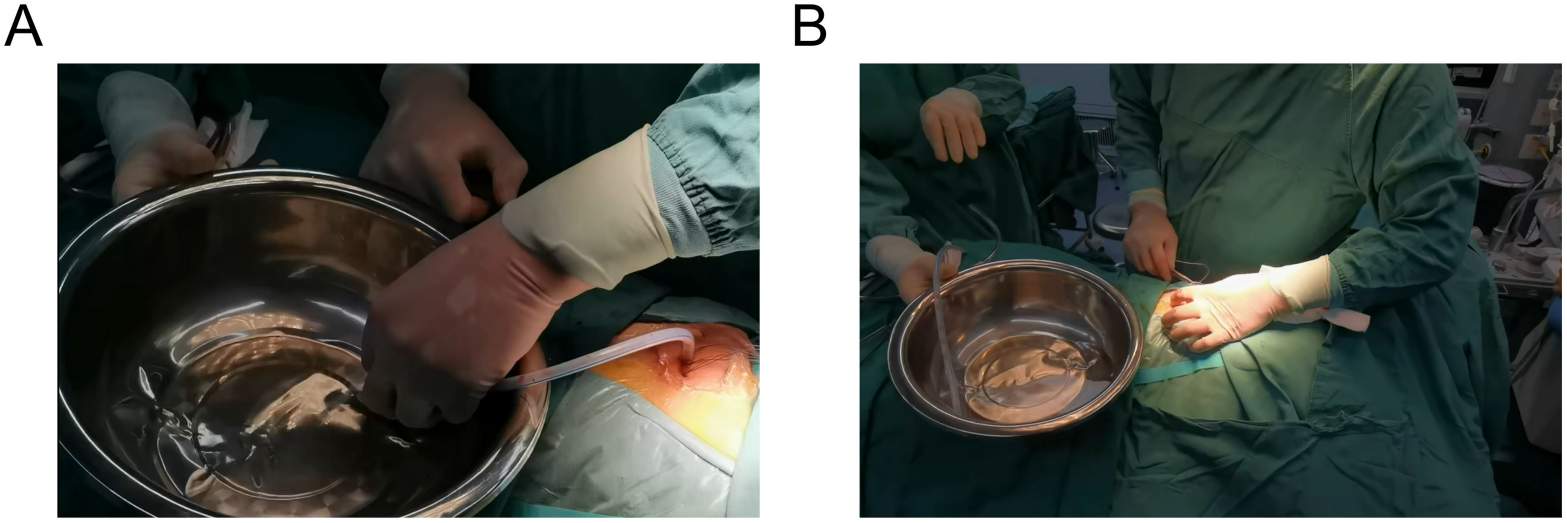
Tubeless procedure images. In the air leakage test, the lung was re-expanded, and a tube was inserted into the water to test whether air leaked **(A)**. If no air leakage was indicated by the absence of bubbles in the water, the chest tube was removed, and the skin was sutured **(B)**.

After lesion resection, the anesthesiologist was instructed to expand the lung completely with 20 cmH_2_O. Afterward, the ventilator parameters were adjusted to provide a positive end-expiratory pressure (PEEP) of 7 cmH_2_O. A chest tube was placed and connected to a negative pressure continuous suction system, after which the muscles and subcutaneous tissues were sutured, and only the chest tube remained. Afterward, the lung was re-expanded with 20 cmH_2_O pressure, and then the tube was inserted into the water to test for air leakage (**Figure 1A**); the duration lasted for 30–60 s. The negativity of the air leakage test was defined as the absence of bubbles in the water; combined with other tubeless eligibility criteria, the chest tube was removed and the skin was sutured (**Figure 1B**). If a single non-fused bubble occurred, it represented mild-degree air leakage; if intermittent fusion bubbles occurred, it represented moderate-degree air leakage; and if persistent fusion bubbles occurred, it represented severe-degree air leakage. If air leakage occurred, no matter the degree, the chest tube remained and the skin was sutured. The air leakage test was commonly performed two to three times: immediately after resection, after intervention if air leakage was detected, and before closing the chest cavity. The tubeless eligibility criteria included the following: There was no air leakage in the air leakage test, no bullous or emphysematous changes in the lung, no dense adhesion of pleura, and no oozing or accumulation of pleural effusion.

For both groups, a bedside chest X-ray was systematically performed on the day of surgery. Three ibuprofen and codeine tablets (0.2 g ibuprofen and 13 mg codeine per tablet) were administered twice a day for postoperative analgesia as needed. In detail, 25 patients (41.7%) in the tubeless group and 47 patients (78.3%) in the chest tube group received postoperative analgesia.

### 2.3 Data collection and follow-up

Data on patients’ characteristics, including age, sex, diagnosis, nodule location, and nodule size, were collected. Perioperative outcomes, including operational time, perioperative blood loss, tube duration, total hospital stay, postoperative hospital stay, and postoperative complications, were also collected. Moreover, pain visual analogue scale (VAS) scores at rest on postoperative day (POD) 1, 2, and 3 were collected.

The patients were followed up by outpatient visits or telephone on month 1 (M1) and month 3 after the surgery day. Post-discharge events were captured during the 3-month follow-up. Moreover, if symptoms occurred, a chest X-ray was performed during the follow-up. Pneumothorax was defined as lung compression greater than 20% on chest X-ray. When a lung compression on the chest X-ray was greater than 30%, a thin chest tube drainage at the second intercostal space was performed.

In addition, the patients’ satisfaction was assessed using a self-reported five-point Likert scale (ranging from one to five points) on M1, among which 1 represented very unsatisfied, 2 represented unsatisfied, 3 represented acceptable, 4 represented satisfied, and 5 represented very satisfied. Moreover, overall satisfaction was calculated by satisfied + very satisfied.

### 2.4 Statistical analysis

Comparisons of data between the tubeless group and the chest tube group were performed using unpaired *t*-tests, Wilcoxon rank sum tests, chi-square tests, or Fisher’s exact tests, as appropriate. Multivariable linear or logistic regressions were performed to adjust the outcomes, as appropriate. SPSS Statistics software (IBM, USA) was used for statistical analysis. Statistical significance was defined as a *P*-value less than 0.05.

## 3 Results

### 3.1 Study flow

**Figure 2:**
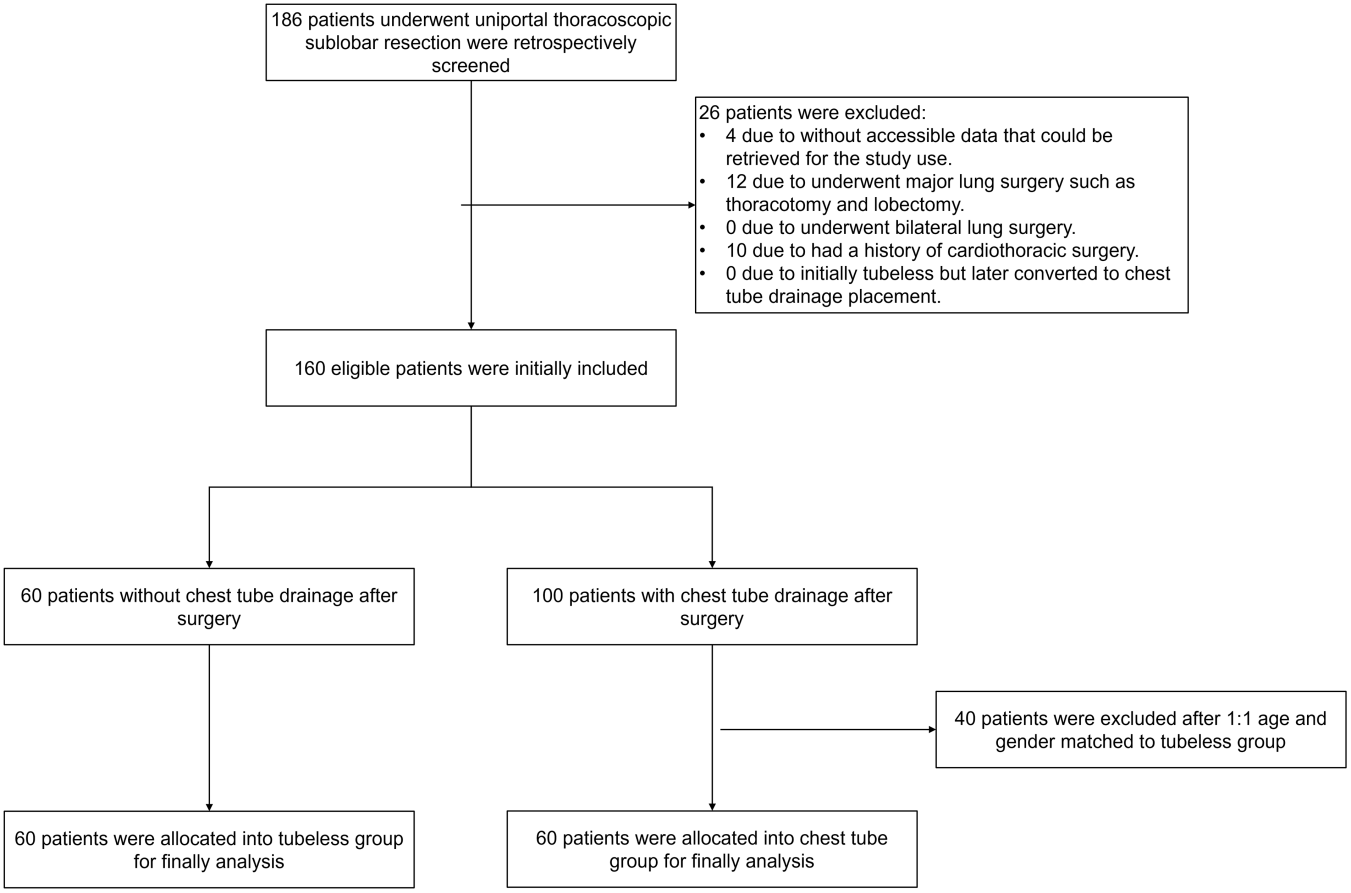
Study flow chart.

A sum of 186 patients who underwent uniportal thoracoscopic sublobar resection were retrospectively screened. Among them, 26 patients were excluded for the following reasons: four due to inaccessible data that could not be retrieved for study use, 12 because they underwent major lung surgery such as thoracotomy and lobectomy, and 10 because they had a history of cardiothoracic surgery. Then, 160 eligible patients were initially included, consisting of 60 patients without chest tube drainage after surgery and 100 patients with chest tube drainage after surgery. The 60 patients without chest tube drainage after surgery were allocated into the tubeless group for final analysis. The 100 patients with chest tube drainage after surgery were proposed to 1:1 age and sex matching to the tubeless group; among them, 40 patients were excluded, and the remaining 60 patients were allocated into the chest tube group for final analysis (**Figure 2**).

### 3.2 Comparison of patients’ characteristics

The mean age was 51.9 ± 12.5 years in the tubeless group, which included 35.0% male and 65.0% female. A total of 73.3%, 16.7%, 1.7%, and 8.3% of the patients in the tubeless group were diagnosed with adenocarcinoma, inflammatory pseudotumor, squamous cell carcinoma, and others, respectively. Moreover, 18.3%, 0.0%, 20.0%, 30.0%, 26.7%, and 5.0% of the patients in the tubeless group had nodules located in the right upper lobe, right middle lobe, right lower lobe, left upper lobe, left lower lobe, and two lobes, respectively, with a nodule size of 11.3 ± 6.0 mm. There were 10.0%, 83.3%, and 6.7% patients in the tubeless group receiving wedge resection, segmentectomy, or combination, respectively (**Table 1**). The mean age of the patients in the chest tube group was 54.2 ± 12.9 years; 36.7% were male, and 63.3% were female. A total of 75.0%, 11.7%, 5.0%, and 8.3% of the patients in the chest tube group had a diagnosis of adenocarcinoma, inflammatory pseudotumor, squamous cell carcinoma, and others, respectively. In addition, 25.0%, 11.7%, 13.3%, 25.0%, 21.7%, and 3.3% of the patients in the chest tube group had nodules located in the right upper lobe, right middle lobe, right lower lobe, left upper lobe, left lower lobe, and two lobes, respectively, and the nodule size was 12.6 ± 8.1 mm. There were 5.0%, 63.3%, and 31.7% patients in the chest tube group receiving wedge resection, segmentectomy, or combination, respectively. After comparison, there were no differences observed in most of the abovementioned characteristics between the tubeless group and the chest tube group (*P* > 0.05), except for surgery type (*P =* 0.002).

**Table 1:** Characteristics of patients.

Items	Chest tube (N = 60)	Tubeless (N = 60)	P-value
Age (years), mean ± SD	54.2 ± 12.9	51.9 ± 12.5	0.330
Sex, n (%)			0.849
Male	22 (36.7)	21 (35.0)	
Female	38 (63.3)	39 (65.0)	
Diagnosis, n (%)			0.673
Adenocarcinoma	45 (75.0)	44 (73.3)	
Inflammatory pseudotumor	7 (11.7)	10 (16.7)	
Squamous cell carcinoma	3 (5.0)	1 (1.7)	
Others	5 (8.3)	5 (8.3)	
Nodule location, n (%)			0.101
Right upper lobe	15 (25.0)	11 (18.3)	
Right middle lobe	7 (11.7)	0 (0.0)	
Right lower lobe	8 (13.3)	12 (20.0)	
Left upper lobe	15 (25.0)	18 (30.0)	
Left lower lobe	13 (21.7)	16 (26.7)	
Two lobes	2 (3.3)	3 (5.0)	
Nodule size (mm), mean ± SD	12.6 ± 8.1	11.3 ± 6.0	0.338
Surgery type, n (%)			0.002
Wedge resection	3 (5.0)	6 (10.0)	
Segmentectomy	38 (63.3)	50 (83.3)	
Combination	19 (31.7)	4 (6.7)	

“Others” in the diagnosis item mainly include malignancies such as small cell lung cancer, alveolar cell cancer, lymphoma, etc.

SD, standard deviation.

### 3.3 Comparison of perioperative outcomes

The operational time was shorter in the tubeless group than in the chest tube group (33.3 ± 11.2 min versus 58.8 ± 27.2 min, *P* < 0.001), and perioperative blood loss was lower in the tubeless group than in the chest tube group [median (interquartile range): 10.0 (5.0–20.0) mL versus 20.0 (10.0–20.0) mL, *P =* 0.007]. Moreover, total hospital stay (6.5 ± 1.6 days versus 7.7 ± 2.2 days, *P =* 0.001) and postoperative hospital stay (3.1 ± 1.1 days versus 4.5 ± 1.7 days, *P* < 0.001) were both shorter in the tubeless group compared to the chest tube group (**Table 2**).

**Table 2:** Perioperative outcomes.

Items	Chest tube (N = 60)	Tubeless (N = 60)	Mean/median/risk difference (95% CI)	P-value
Operational time (min), mean ± SD	58.8 ± 27.2	33.3 ± 11.2	-25.6 (-33.1 to -18.1)	<0.001
Perioperative blood loss (mL), median (IQR)	20.0 (10.0–20.0)	10.0 (5.0–20.0)	-10.0 (-10.0–0.0)	0.007
Tube duration (days), mean ± SD	2.6 ± 1.4	–	–	–
Total hospital stay (days), mean ± SD	7.7 ± 2.2	6.5 ± 1.6	-1.2 (-1.9 to -0.5)	0.001
Postoperative hospital stay (days), mean ± SD	4.5 ± 1.7	3.1 ± 1.1	-1.4 (-1.9 to -0.8)	<0.001
Postoperative complications, n (%)				
Pneumothorax	0 (0.0)	1 (1.7)	0.017 (-0.016~0.050)	0.500
Pleural effusion	0 (0.0)	0 (0.0)	0.000 (0.000~0.000)	1.000
Pulmonary infection	0 (0.0)	0 (0.0)	0.000 (0.000~0.000)	1.000
Prolonged air leak	0 (0.0)	0 (0.0)	0.000 (0.000~0.000)	1.000
Subcutaneous emphysema	0 (0.0)	0 (0.0)	0.000 (0.000~0.000)	1.000
Atelectasis	0 (0.0)	0 (0.0)	0.000 (0.000~0.000)	1.000
Hemothorax	0 (0.0)	0 (0.0)	0.000 (0.000~0.000)	1.000
Arrhythmia	0 (0.0)	0 (0.0)	0.000 (0.000~0.000)	1.000
Need for reintervention	0 (0.0)	1 (1.7)	0.017 (-0.016~0.050)	0.500
ICU transfer	0 (0.0)	0 (0.0)	0.000 (0.000~0.000)	1.000
Readmission	0 (0.0)	1 (1.7)	0.017 (-0.016~0.050)	0.500
30-day mortality	0 (0.0)	0 (0.0)	0.000 (0.000~0.000)	1.000
Conversion to chest tube drainage, n (%)	–	1 (1.7)	–	–

Mean difference (95% CI) is presented for operational time, total hospital stay, and postoperative hospital stay. Median difference (95% CI) is presented for perioperative blood loss. Risk difference (95% CI) is presented for postoperative complications and conversion to chest tube drainage.

SD, standard deviation; IQR, interquartile range; ICU, intensive care unit; CI, confidence interval.

Notably, only one (1.7%) patient experienced pneumothorax in the tubeless group, which was not significantly different from the 0 (0.0%) patient in the chest tube group (*P =* 0.500). Meanwhile, the one (1.7%) patient in the tubeless group in whom pneumothorax occurred was subsequently readmitted and received chest tube drainage, which was considered as a conversion. No patient experienced other postoperative complications such as pleural effusion, pulmonary infection, subcutaneous emphysema, 30-day mortality, etc., in both groups (**Table 2**).

**Figure 3:**
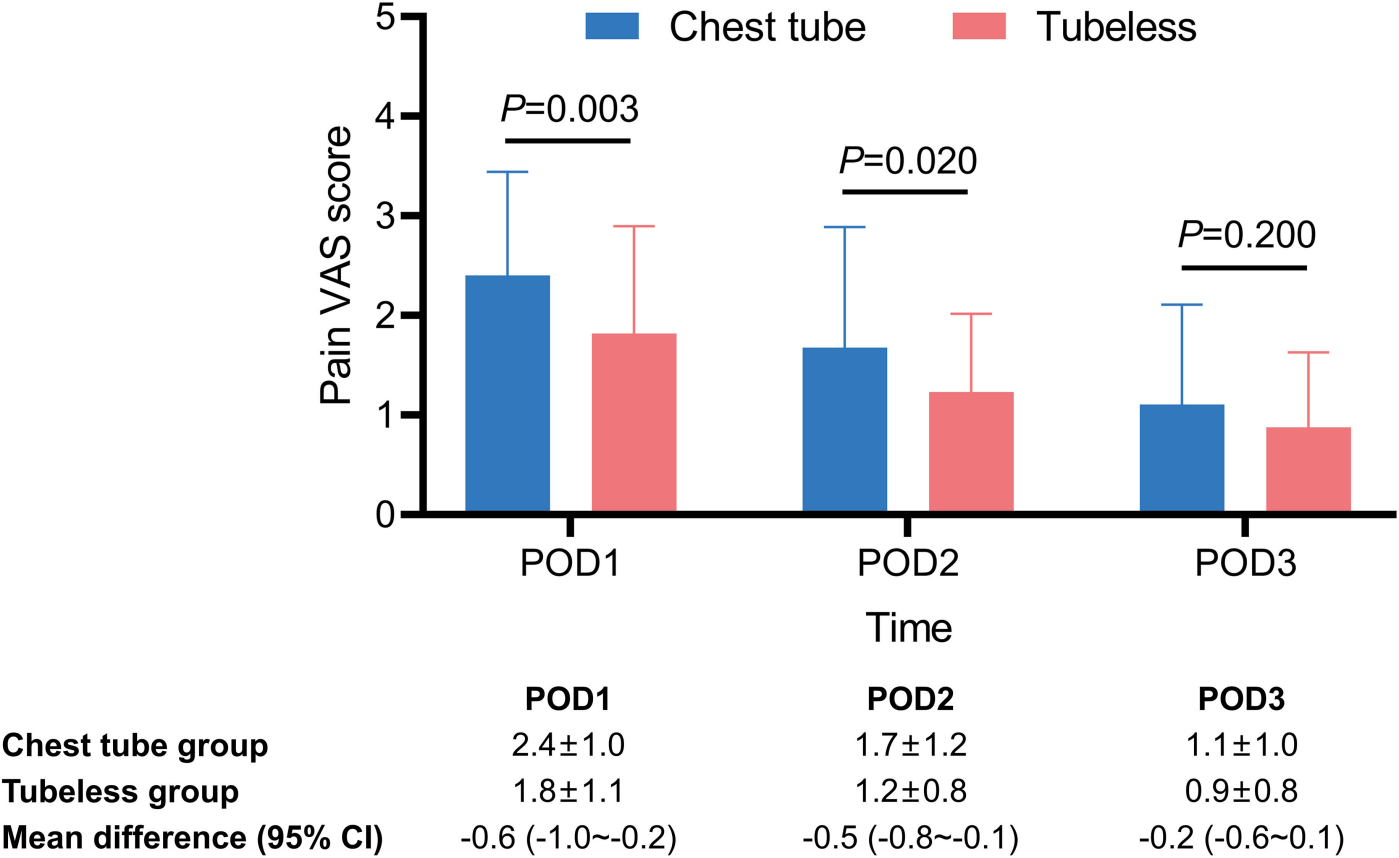
Pain VAS score assessment on POD1, POD2, and POD3.

In addition, the VAS pain score was lower in the tubeless group compared with the chest tube group on POD1 (1.8 ± 1.1 versus 2.4 ± 1.0, *P =* 0.003) and POD2 (1.2 ± 0.8 versus 1.7 ± 1.2, *P =* 0.020) but was not significantly different between them on POD3 (0.9 ± 0.8 versus 1.1 ± 1.0, *P =* 0.200) (**Figure 3**).

### 3.4 Comparison of patients’ satisfaction

In the tubeless group, 38.3%, 36.7%, and 25.0% of the patients reported being very satisfied, satisfied, and acceptable, respectively, and no patient reported being unsatisfied or very unsatisfied. In the chest tube group, 33.3%, 48.3%, and 18.3% of the patients were very satisfied, satisfied, and acceptable, respectively, and no patient was unsatisfied or very unsatisfied, respectively. After comparison, the satisfaction scale score did not differ between the tubeless group and the chest tube group (*P =* 0.410). After categorization of very satisfied and satisfied into overall satisfaction, it was observed that overall satisfaction was also not different between the tubeless group and the chest tube group (75.0% versus 81.7%, *P =* 0.375) (**Table 3**).

**Table 3:** Satisfaction assessment at M1.

Items	Chest tube (N = 60)	Tubeless (N = 60)	P-value
Satisfaction scale, n (%)			0.410
Very satisfied	20 (33.3)	23 (38.3)	
Satisfied	29 (48.3)	22 (36.7)	
Acceptable	11 (18.3)	15 (25.0)	
Unsatisfied	0 (0.0)	0 (0.0)	
Very unsatisfied	0 (0.0)	0 (0.0)	
Overall satisfaction, n (%)			0.375
Yes	49 (81.7)	45 (75.0)	
No	11 (18.3)	15 (25.0)	

Overall satisfaction was defined as very satisfied + satisfied.

M1, month 1.

### 3.5 Outcomes after adjustment

After adjustment using multivariable linear or logistic regression analyses, tubeless vs. chest tube was independently correlated with a lower operational time (*β* = -0.482, *P* < 0.001), total hospital stay (*β* = -0.275, *P =* 0.003), postoperative hospital stay (*β* = -0.425, *P* < 0.001), pain VAS score on POD1 (*β* = -2.639, *P =* 0.009), and pain VAS score on POD2 (*β* = -2.062, *P =* 0.042) but was not independently associated with perioperative blood loss (*β* = -0.142, *P =* 0.110), pain VAS score on POD3 (*β* = -0.944, *P =* 0.348), pneumothorax [odds ratio (OR) = 4.811 × 10^7^, *P =* 0.997], nor overall satisfaction (OR = 0.735, *P =* 0.553) (**Table 4**).

**Table 4:** Adjustment by multivariable linear or logistic regression analyses.

Outcomes	Parameter	β/OR	P-value
Multivariable linear regression			
Operational time	Tubeless vs. chest tube	-0.482	<0.001
Perioperative blood loss	Tubeless vs. chest tube	-0.142	0.110
Total hospital stay	Tubeless vs. chest tube	-0.275	0.003
Postoperative hospital stay	Tubeless vs. chest tube	-0.425	<0.001
Pain VAS score on POD1	Tubeless vs. chest tube	-2.639	0.009
Pain VAS score on POD2	Tubeless vs. chest tube	-2.062	0.042
Pain VAS score on POD3	Tubeless vs. chest tube	-0.944	0.348
Multivariable logistic regression			
Pneumothorax	Tubeless vs. chest tube	4.811 × 107	0.997
Overall satisfaction	Tubeless vs. chest tube	0.735	0.533

Adjustment was performed by multivariable linear or logistic regressions, including the following parameters: tubeless vs. chest tube, age, sex, diagnosis, nodule location, nodule size, and surgery type. Multivariable linear regression data are presented using β and *P*-value, while multivariable logistic regression data are presented using OR and *P*-value.

VAS, visual analogue scale; POD, postoperative day; OR, odds ratio.

## 4 Discussion

To improve the postoperative complications caused by chest tube placement, VATS without chest tube drainage is proposed for some specifically selected patients. In a previous study, pulmonary nodule patients who underwent unilateral wedge resection by uniportal VATS but without severe pleural adhesion, emphysema, lung infection, perioperative anticoagulant administration, segmentectomy, or lobectomy were selected, and the degree of simplicity of the operation and the degree of air leakage during surgery were assessed to perform the procedure without chest tube drainage (12). In another study, patients whose lung lesions were ≤2 cm, individual unilateral wedge resection was ≤3, air leak test was negative during surgery, and did not experience severe pleural adhesion, bleeding, or the administration of anticoagulant or antiplatelet drugs were chosen to undergo uniportal thoracoscopic wedge resection without chest tube drainage (13). Moreover, one study focused mainly on intraoperative air leakage test results, taking into account the tendency toward intrathoracic adhesion bleeding, to perform thoracoscopic major lung resection without chest tube drainage (19).

The pleural drainage decision-making strategy is important, especially the patient selection, intraoperative air leak assessment, and post-operative surveillance strategies (20). Patient selection for chest drainage follows the “right tube, right system, right criteria” principle, matching catheter caliber to the pleural condition (e.g., ≤14 Fr for spontaneous pneumothorax, 19–24 Fr for routine lobectomy, and larger tubes for high-risk hemothorax or thick empyema). Intraoperative air leak assessment is qualitative with analog systems but objectively quantified by digital devices using sustained low airflow (20–40 mL/min) to guide removal. Postoperative surveillance prioritizes early transition to water seal or low-regulated “seal” mode, standardized removal thresholds (≤300–500 mL/day of non-bloody fluid or ≤20–40 mL/min airflow with full lung expansion), and protocolized escalation (e.g., rTPA/DNase, VATS) for retained collections or persistent leaks, supported by ultrasound and multidisciplinary protocols to minimize complications and enhance recovery (20).

Our institution screened patients with small intraoperative wounds and a limited resection range, without bilateral lesion or cardiothoracic surgery history, and then filtered them using tubeless eligibility criteria, including no air leakage in the air leakage test, no bullous or emphysematous changes in the lung, no dense adhesion of pleura, and no oozing or accumulation of pleural effusion, to perform uniportal thoracoscopic sublobar resection without chest tube drainage, which referred to the previous studies (12–15, 19), expert consensus (7), and our clinical experience. These patients who underwent uniportal thoracoscopic sublobar resection without chest tube drainage served as the tubeless group, while others with chest tube drainage served as the chest tube group. This study found that the operation time and perioperative blood loss were lower in the tubeless group than in the chest tube group; these findings might be due to the less complexity of tubeless strategy compared to chest tube drainage. Moreover, perioperative blood loss was minimal in both groups, with a median value of 10.0 mL in the tubeless group and 20.0 mL in the chest tube group, which were in line with the findings of previous studies (12, 14).

Pneumothorax risk is a major consideration for VATS without chest tube drainage. Two earlier studies reported a higher incidence of pneumothorax in patients who underwent thoracoscopic wedge resection without chest tube drainage than in those who underwent chest tube drainage (16, 21). Notably, after the advancements in VATS technology and screening criteria for selected patients were implemented, an increasing number of recent studies reported a similar incidence of pneumothorax in patients who underwent thoracoscopic wedge resection or major lung surgeries without chest tube drainage compared with those with chest tube drainage, and some studies reported that no pneumothorax occurred in patients without chest tube drainage. Moreover, the incidences of other perioperative complications did not differ between patients who did not undergo chest tube drainage and those who underwent chest tube drainage (12, 14, 15, 19, 22). This study revealed that the incidence of pneumothorax did not differ between uniportal thoracoscopic sublobar resection patients in the tubeless group and those in the chest tube group, nor did the incidence of other complications such as pleural effusion and pulmonary infection, which is in line with the findings of previous studies (12, 14, 15, 19, 22). These findings might be due to the fact that after selecting feasible patients via a tubeless eligibility criteria, including no air leakage in the air leakage test, no bullous or emphysematous changes in the lung, no dense adhesion of pleura, and no oozing or accumulation of pleural effusion, the patients were at a low risk of pneumothorax or other complications without chest tube drainage.

Regarding postoperative pain assessment, both earlier and recent studies have consistently shown that, compared with chest tube drainage, VATS without chest tube drainage reduces postoperative pain, especially short-term pain (12, 14, 16, 19, 21). In this study, pain on POD1 and POD2 was lower in uniportal thoracoscopic sublobar resection patients in the tubeless group than in those in the chest tube group. This finding may be due to direct pain caused by the presence of a chest tube and restricted movement by the chest tube.

Moreover, compared with patients with chest tube drainage, patients with VATS without chest tube drainage have shorter hospital stays (12, 14, 16, 22)—for example, a previous study reported that the postoperative length of stay was shorter in patients who underwent uniportal thoracoscopic wedge resection without chest tube drainage than in those who underwent chest tube drainage (12). Another study involving 12 years of clinical experience revealed that hospitalization duration was less prolonged in patients who underwent thoracoscopic wedge resection without chest tube drainage than in those who underwent chest tube drainage both before and after adjustment (22). This study revealed that the total hospital stay and the postoperative hospital stay were both shorter in uniportal thoracoscopic sublobar resection patients in the tubeless group than in those in the chest tube group. These findings might be due to the chest tube maintenance time in the chest tube group and the relatively fast recovery in the tubeless group. In addition, the patients’ satisfaction might be comparable between uniportal thoracoscopic sublobar resection patients in the tubeless group and the chest tube group, which might have resulted from the comparable perioperative outcomes and patient recovery after the surgery itself.

Some limitations of this study should be mentioned. First, owing to the retrospective cohort design, selection bias exists in this study, and a further randomized, controlled study is needed for further validation. Second, the sample size was somewhat limited, leading to a lack of generalizability of the results; thus, a large-sample study could be considered in the future for verification. Third, only adults were included in this study, and fewer elderly individuals were included; therefore, the tubeless strategy in elderly patients should be evaluated in future studies. Fourth, the satisfaction scale was self-reported and not previously validated in the specific patient type in this study; therefore, further validation on this point should be explored.

## 5 Conclusion

In conclusion, uniportal thoracoscopic sublobar resection without chest tube drainage may be feasible in a selected cohort of low-risk patients without intraoperative air leakage. However, further validation using prospective studies with standardized outcome capture is still needed.

## Data Availability

The original contributions presented in the study are included in the article/supplementary material. Further inquiries can be directed to the corresponding author.
